# Current status and implications of microRNAs in ovarian cancer diagnosis and therapy

**DOI:** 10.1186/1757-2215-5-44

**Published:** 2012-12-13

**Authors:** Mohd Saif Zaman, Diane M Maher, Sheema Khan, Meena Jaggi, Subhash C Chauhan

**Affiliations:** 1 Cancer Biology Research Center, Sanford Research/USD, 2301 East 60th Street North, Sioux Falls, SD 57104, USA; 2 Department of Obstetrics and Gynecology, Sanford School of Medicine, The University of South Dakota, 2301 East 60th Street North, Sioux Falls, SD 57105, USA; 3 Basic Biomedical Science Division, Sanford School of Medicine, The University of South Dakota, 2301 East 60th Street North, Sioux Falls, SD 57105, USA

**Keywords:** Ovarian cancer, miRNAs, Chemoresistance, Diagnosis, Prognosis, Therapy, miR-200, Let-7

## Abstract

Ovarian cancer is the fifth most common cancer among women and causes more deaths than any other type of female reproductive cancer. Currently, treatment of ovarian cancer is based on the combination of surgery and chemotherapy. While recurrent ovarian cancer responds to additional chemotherapy treatments, the progression-free interval becomes shorter after each cycle, as chemo-resistance increases until the disease becomes incurable. There is, therefore, a strong need for prognostic and predictive markers to help optimize and personalize treatment in order to improve the outcome of ovarian cancer. An increasing number of studies indicate an essential role for microRNAs in ovarian cancer progression and chemo-resistance. MicroRNAs (miRNAs) are small endogenous non-coding RNAs (~22bp) which are frequently dysregulated in cancer. Typically, miRNAs are involved in crucial biological processes, including development, differentiation, apoptosis and proliferation. Two families of miRNAs, miR-200 and let-7, are frequently dysregulated in ovarian cancer and have been associated with poor prognosis. Both have been implicated in the regulation of epithelial-to-mesenchymal transition, a cellular transition associated with tumor aggressiveness, tumor invasion and chemo-resistance. Moreover, miRNAs also have possible implications for improving cancer diagnosis; for example miR-200 family, let-7 family, miR-21 and miR-214 may be useful in diagnostic tests to help detect ovarian cancer at an early stage. Additionally, the use of multiple target O-modified antagomirs (MTG-AMO) to inhibit oncogenic miRNAs and miRNA replacement therapy for tumor suppressor miRNAs are essential tools for miRNA based cancer therapeutics. In this review we describe the current status of the role miRNAs play in ovarian cancer and focus on the possibilities of microRNA-based therapies and the use of microRNAs as diagnostic tools.

## Introduction

Epithelial ovarian cancer (referred to as ovarian cancer in this review) is the fifth most common cancer among women and causes more deaths than any other type of female reproductive cancer [1]. Signs and symptoms of ovarian cancer are frequently absent or ambiguous early on and due to a lack of early detection strategies most (>60%) patients are diagnosed with advanced-stage disease. The five year survival rate is less than 30% for these advanced-stage patients and, despite advances in chemotherapy, survival rates have only modestly improved over the past 40 years [1-3]. Pathologically, ovarian cancer is a heterogeneous disease comprised of serous, mucinous, clear cell, and endometrioid subtypes. Serous tumors are the most common subtype. Each subtype is associated with diverse genetic risk factors and molecular events during oncogenesis and is characterized by distinct mRNA expression profiles. It has been observed that subtypes respond differently to chemotherapy. The response rate of clear cell carcinomas (15%) is very low, whereas the response rate for high-grade serous is 80%, resulting in a lower 5-year survival for clear cell compared with high-grade serous carcinoma in patients with advanced stage tumors (20% versus 30%) [4,5].

The standard treatment for advanced ovarian cancer is surgical tumor debulking (removal of all tumor and metastasis that can be macroscopically detected in the entire abdomen region), followed by platinum-based chemotherapy [6]. Neoadjuvant therapy, the use of chemotherapy or radiation prior to surgery, may be an option for patients with stage IIIC or IV ovarian cancer which typically presents with a large tumor burden and extensive metastases. For these patients optimal debulking may be difficult to achieve, making neoadjuvant therapy an important option [7]; however, there is currently an ongoing debate to clearly identify which patients would benefit from neoadjuvant therapy [8,9].

Chemotherapy in ovarian cancer includes platinum-based drugs, cisplatin or carboplatin coupled with paclitaxel. After first-line treatment with carboplatin and paclitaxel, most patients eventually relapse with a median progression-free survival of 18 months. Recurrent ovarian cancer initially responds to additional chemotherapy; however, the progression-free interval becomes shorter after each cycle as chemo-resistance increases until the disease becomes incurable [10]. A number of molecular mechanisms have been characterized to explain the development of resistance to chemotherapy, such as increased DNA repair activity and defective DNA damage response [11], increased anti-apoptotic regulator activity [12,13], growth factor receptor deregulation [14][15] and post-translational modification or aberrant expression of β-tubulin and other microtubule regulatory proteins [16].

**Figure 1:**
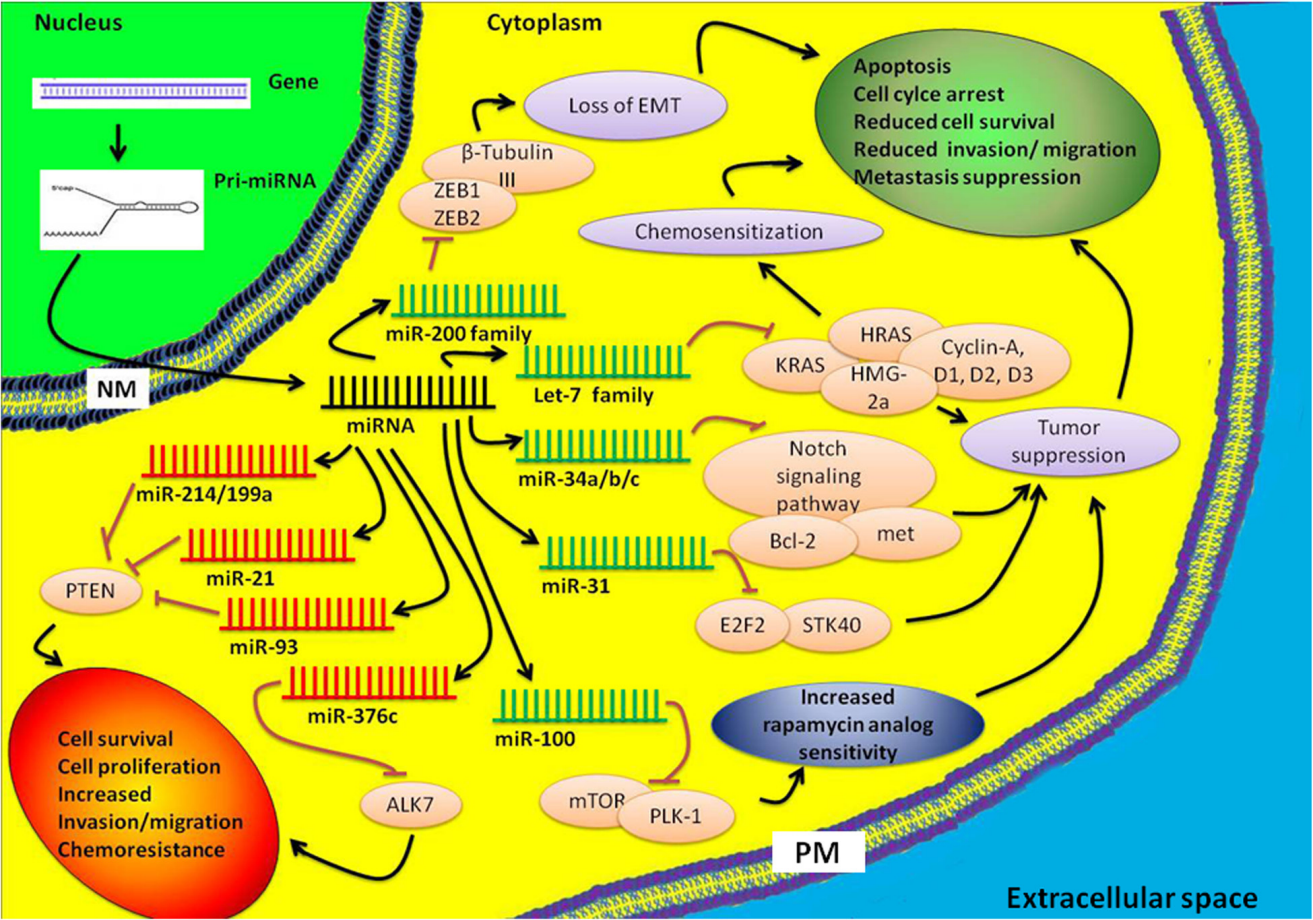
**Oncogenic and tumor suppressor miRNAs in ovarian carcinoma.** Based on their function miRNAs can be used for diagnostics and therapeutics. Certain miRNAs such as miR-200 family, let-7 family, miR-21, miR-214 and miR-100 have strong diagnostic/prognostic potential in ovarian cancer. Use of antagonists for oncogenic microRNAs and microRNA replacement therapy for tumor suppressor miRNAs are important tools in miRNA based cancer treatment. EMT-Epithelial to Mesenchymal Transition; NM-Nuclear membrane; PM-Plasma membrane.

The recently discovered microRNAs (miRNAs) constitute a novel regulatory layer of gene expression and have been implicated in the etiology of various kinds of human cancers. miRNAs are small (~22bp) endogenous non-coding RNAs and are frequently dysregulated in cancer. Their role is to modulate gene expression mainly by base-pairing to the 3’-UTR (untranslated region) of the target mRNA, eventually causing either translational repression, mRNA cleavage, or destabilization [17]. In ovarian carcinoma the expression of various miRNAs has been found to be dysregulated [18]. Recent reports support a role for miRNAs in the initiation and progression of ovarian carcinoma [19-21] by promoting the expression of proto-oncogenes or by inhibiting the expression of tumor suppressor genes. In this review we focus on the current status of the role miRNAs play in ovarian cancer (Figure-1 and Table-1). We also describe their diagnostic / prognostic and therapeutic potential. 

**Table 1:** miRNAs: functions and targets in ovarian cancer

**microRNAs**	**Function**	**Targets**	**Reference**
miR-200 family	Tumor suppressor	ZEB1,ZEB2,β tubulin III	[31,42,45,47,48,51-59,111,112]
	(Loss of EMT)		
Let-7 family	Tumor suppressor	KRAS,HRAS,C-MYC,HMGA-2,CyclinA,D1,D2,D3,CDC25,CDK6	[47,68-81,110,113,116,117]
	(Chemosensitization)		
miR-34a/b/c	Tumor suppressor	c-myc,CDK6,Notch-1,MET,E2f3,Bcl2,cyclinD1	[82,119]
	(Reduced invasion/migration/proliferation)		
miR-100	Tumor suppressor	mTOR, PLK-1	[83-85]
	(Increased sensitivity to rapamycin analogs)		
miR-31	Tumor suppressor	E2F2, STK40	[86]
	(Increased apoptosis)		
miR-214/199a*	Oncogenic	PTEN	[51,87-91,93,112]
	(Chemoresistance)		
miR-376c	Oncogenic	ALK7	[21,98]
	(Chemoresistance)		
miR-93	Oncogenic	PTEN	[100-102]
	(Chemoresistance)		
miR-21	Oncogenic	PTEN	[47,51,104-107,112]
	(Chemoresistance)		

miRNAs in ovarian carcinoma can either be oncogenic or tumor suppressor. Tumor suppressor miRNAs suppress oncogenes resulting in either loss of EMT (Epithelial Mesenchymal Transition), chemosensitization or tumor suppression. Whereas, oncogenic miRNAs target tumor suppressor genes leading to chemo resistance and reduced survival.

### miRNAs

miRNAs were first discovered in 1993 by Lee, Feinbaum and Ambros in the nematode *C. elegans*[22] and are now known to be present and highly conserved among a wide range of species [23]. Mature miRNAs are derived from precursors called pri-miRNAs, composed of hundreds or thousands of nucleotides [17,24,25]. miRNAs precursor sequences are located in different parts of nuclear DNA and may constitute mono- or policistrone transcriptional units. Pri-miRNAs are transcribed mainly by polymerase RNA II. Subsequently, they are cleaved by the endonuclease Drosha and cofactor DGCR8 into a structure known as precursor miRNA or pre-miRNA. Pre-miRNAs, ~60 nucleotide stem-loop molecules, are transported from the nucleus to the cytoplasm by Exportin 5 and protein Ran-GTP and further processed by the Dicer enzyme into a ~22 nucleotide double-stranded microRNA [26]. The double-stranded miRNA assembles into a ribonucleoprotein complex which is known as the RNA induced silencing complex (RISC) [27]. The RISC induces unwinding of the double-stranded molecule into single stranded miRNA, concomitantly degrading the complementary strand. In animals the miRNA–RISC binds to 3’ untranslated region (3’ UTR) of mRNA, does not require perfect complementarity, and induces inhibition of translation at the initiation or elongation phase [27]. The mode of inhibition may depend, in part, on the level of complementarity of the miRNAs where perfect or near perfect complementarity favors degradation. The mechanism of 3’UTR mRNA target regulation is complex. Nevertheless, recent studies suggest that it is a two step process in which inhibition of translation is done first, followed by mRNA decay due to deadenylation of the mRNA [28]. The seed sequence is essential for the binding of the miRNA to the mRNA. The seed sequence is a conserved heptamerical sequence which is mostly situated at positions 2–7 from the 5’ end of the miRNA, although other factors are also important [17,29]. Gu et. al. [30] have suggested that miRNA target sites can also be found in the 5’ UTR or even in the coding region of the mRNA. By binding to 5’ UTR sequences miRNAs can also activate translation. Thus, inhibition of posttranscriptional mRNA processing is not the only way of regulating miRNA-dependent gene expression [31]. Moreover, as miRNAs do not require perfect complementarity for functional interactions with mRNA targets, a single miRNA can regulate multiple targets and conversely, multiple miRNAs are known to regulate individual mRNAs [32].

### miRNAs in cancer

miRNAs are involved in crucial biological processes, including development, differentiation, apoptosis and proliferation [33]. This is done through imperfect pairing with target messenger RNAs (mRNAs) of protein-coding genes and the transcriptional or post-transcriptional regulation of their expression [26]. The first reported link between miRNAs and cancer regarded chronic lymphocytic leukemia, wherein miR-15 and miR-16 were found to be deleted or down-regulated in a majority of tumors [34]. Since then, changes in the expression level of miRNAs have subsequently been detected in many types of human tumors [35]. miRNAs have various roles in oncogenesis as they can function either as tumor suppressors (*e.g.*, miR-15a and miR-16-1) or oncogenes (*e.g.*, miR-155 or members of the miR-17–92 cluster). Recent studies on the abnormal expression of miRNAs in cancer have described the following reasons for differential expression: chromosomal rearrangements [36-38], genomic copy number change [39,40], epigenetic modifications [41,42], defects in miRNA biogenesis pathway [43], and regulation by transcription factors [44].

### miRNAs in ovarian cancer

One of the seminal studies done on epithelial ovarian cancer (EOC) and miRNA was done by Zhang et. al. in 2008 [45]. In this study the authors utilized an integrative genomic approach to study miRNA deregulation in human epithelial ovarian cancer. They compared mature miRNA expression profiles in 18 ovarian cancer cell lines and 4 immortalized, non neoplastic cell lines derived from normal ovarian surface epithelium. Thirty five miRNAs were found to be differentially expressed between the two groups of cells. Out of these 35, only 4 were up-regulated, whereas the rest were mostly down-regulated in cancer cells as compared to immortalized non-neoplastic cell lines. The 4 up-regulated miRNAs were miR-26b, miR-182, miR-103 and miR-26a. The list of down-regulated miRNAs included prominent tumor suppressors such as let-7d and miR-127. This showed that miRNA expression profiles can distinguish malignant from nonmalignant ovarian surface epithelium. Zhang et. al. went on to analyze 106 primary human ovarian cancer specimens of various stages and grades using miRNA microarrays. They found that all tumor suppressor miRNA alterations were related to down-regulation in late stage tumors, which included miR-15a, miR-34a and miR-34b. The authors also observed that DNA copy number loss and epigenetic silencing are mainly responsible for down-regulation of the miRNAs. In the case of over-expressed miRNAs (oncogenic miRNAs such as miR-182) the chromosomal regions were found to be amplified in a significant number of cancer samples. Additionally, epigenetic alterations reduced the expression of 16 out of 44 miRNAs which were down-regulated in late-stage ovarian cancer as the expression of these miRNAs, e.g. miR-34b, was restored by DNA demethylation or histone deacetylase inhibiting agents.

In addition several studies have compared the expression profile of miRNAs in a large number of clinical ovarian cancer samples to normal ovarian tissues, ovarian epithelial cell lines or fallopian tubes [42,46-51]. The following sections provide information regarding miRNAs that are suggested to be involved in the pathobiology of ovarian cancer (Tables 1 and 2). 

**Table 2:** miRNA profile of subtypes of ovarian cancer

**Type of ovarian cancer**	**Up-regulated miRNA**	**Down-regulated miRNA**	**References**
Serous	miR-200a, miR-200b, miR-200c, miR-141, miR-93, miR-21,miR-519a, miR-214	let7-b, miR-99a, miR-125b, miR-22, miR-31, miR-34a/b/c	[42,47,51,86,102,106,108,109]
Clear Cell	miR-519a, miR-182, miR-30a,miR-21, miR-200a, miR-200c	miR-100, miR-22, miR-34a/b/c, miR-214	[42,51,83,109]
Mucinous	miR-153, miR-485-5p		[109]
Endometrioid	miR-200a, miR-200b, miR-200c, miR-141		[42]

Most of the miRNA profiling has been done on the serous and clear cell ovarian carcinoma. miR-200 family stands out as the up-regulated miRNA in most types of ovarian cancer. miR-100 plays a specific role in clear cell ovarian carcinoma.

### miRNA-200 family

The miR-200 family contains miR-200a, miR-200b, miR-200c, miR-141 and miR-429 which are arranged in 2 clusters in the human genome. miR-200a, miR-200b and miR-429 are located on chromosome 1, while miR-200c and miR-141 are on chromosome 12 [52]. Iorio et. al. [42] have shown that the miR-200 family is among the most significantly over-expressed miRNAs in epithelial ovarian cancer. The expression of miR-200a and miR-200c was found to be up-regulated in three types of ovarian cancer: serous, endometrioid and clear cell. However, miR-200b and miR-141 are up-regulated in endometrioid and serous subtypes. The role of the miR-200 family in ovarian carcinoma is complicated. While miR-200 family members are believed to be metastasis suppressants, the majority of studies done on the family relate to over expression in ovarian cancer. However, some studies report that miR-200 family members are either down regulated [48] or even unchanged [45]. These differing results may occur because of the use of different normal controls or the inclusion of ovarian stromal cells which lack miR−200 expression.

Further complicating the potential roles of the miR-200 family members, recent studies have implicated the miR-200 family with the regulation of the epithelial to mesenchymal transition (EMT). EMT is a process where epithelial tumor cells are stimulated by extracellular cytokines, e.g. TGFβ, or intracellular molecules such as oncogenic Ras, to change their epithelial characteristics into a mesenchymal phenotype with increased migratory and invasive capabilities. EMT is induced by a group of transcriptional repressors, such as Snail, Slug, TWIST, Id2, ZEB1 and ZEB2. The protein levels of these repressors increase during EMT, resulting in the down-regulation of genes such as E-cadherin which are responsible for the epithelial identity of the cells [53]. The E-cadherin molecules mediate cell-cell adhesion. Park et. al. [54] have shown a positive correlation in the expression of E-cadherin with the expression of miR-200c in ovarian cancer tissues. The miR-200 family members have also been shown to suppress the expression of ZEB1 and ZEB2, thereby suppressing EMT. Over expression of miR-200 a/b/c and/or miR-141 down regulates ZEB1/2 levels, and leads to higher levels of E-cadherin and an epithelial phenotype. On the contrary, ZEB1/2 can inhibit the expression of miR-200 family members by binding to the promoter of both miR-200 clusters thereby blocking transcription. The mechanistic explanation of the above process can be summarized as follows: activation of a trigger such as TGF-β or PDGF-D [52], leads to increased levels of ZEB1/2, decreased expression of miR-200 and the induction of EMT [31,54-57]. The miR-200 family might be down regulated when cancer cells acquire invasive properties and then become up-regulated when the cells undergo mesenchymal to epithelial transition during the process of re-epithelialization, which is evident from the positive correlation of miR-200 and E-cadherin expressions [58].

In a recent study, Leskela et. al. [59] demonstrated the role of the miR-200 family members in controlling β-tubulin III expression and its association with paclitaxel-based treatment response and progression-free survival in ovarian cancer patients. Previous studies demonstrated that high tumoral β-tubulin III expression has been associated with decreased survival with non-small cell lung cancer [60,61], breast [62], head and neck [63] and ovarian cancer [64]. Moreover, a number of studies have also shown that high expression of β-tubulin III is associated with worse treatment response in ovarian cancer [65-67]. Leskela et. al. found that tumors with high levels of β-tubulin III protein have significantly decreased miR-200 expression. They observed the strongest associations with miR-141, miR-429 and miR-200c. miR-200c expression was statistically significantly associated with response to treatment as patients who did not achieve a complete clinical response had lower levels of miR-200c as compared to those showing a complete response. Low expression of miR-200c was also associated with recurrence of ovarian cancer. Moreover, miR-429 expression was found to be significantly associated with recurrence-free survival and overall survival of the patients [59].

### Let-7 family

The let-7 (lethal-7) family in humans consists of 13 miRNAs located on nine different chromosomes [68,69]. In multiple human cancers expression of the let-7 family is significantly reduced. Low let-7 expression has been found to be associated with poor survival of cancer patients [70,71]. Let-7 suppresses multiple ovarian cancer oncogenes, which includes *KRAS*, *HRAS*[72], *c-MYC*[72] and *HMGA-2*[73]. Moreover, it also inhibits cell cycle regulators such as CDC25, CDK6 as well as Cyclin A, D1, D2 and D3 [74,75]. The mechanism of down- regulation for let-7 miRNAs is through copy-number alterations [76]. The genomic locus containing let-7a-3/let-7b was deleted in 44% of ovarian cancer samples studied. Restoration of let-7b expression significantly reduced ovarian tumor growth both *in vitro* and *in vivo*.

Additionally, recent studies have shown a correlation between loss of let-7 and resistance to either chemotherapeutic drugs or radiation [71,77-79]. Using a drug resistant ovarian cancer cell line, Boyerinas et. al. [80] demonstrated that drug sensitivity to taxanes is increased upon over expression of let-7g as it inhibits IMP-1, an RNA binding protein which stabilizes the mRNA of a number of target genes, including, MDR1 (multidrug resistance-1). MDR1 is a member of the adenosine triphosphate binding cassette transporters (ABC transporter family) which pump drugs across the cell membrane to the extracellular space. Therefore, the expression of let-7g resulted in a decrease in MDR1 and sensitized the cells to Taxane treatment.

On the other hand, Lu et. al. [81] observed that ovarian cancer patients responding to a regimen of platinum and paclitaxel had significantly lower let-7a expression than those who did not respond to treatment. Moreover, survival data indicated that patients with high let-7a survived better when they were treated with platinum only (no paclitaxel) as compared to those having low expression of let-7a. The authors conclude that miRNA-let-7a expression can be a potential marker for selection of chemotherapeutic agents in ovarian cancer treatment.

### miRNA-34 a/b/c

Quantitative-RT PCR and *in-situ* hybridization in a panel of 83 human ovarian cancer samples showed a significant decrease in miR-34a/b/c expression. The decrease was also correlated with the p53 status as p53 regulates the expression of miR-34 family members by promoter methylation and copy number alterations [82]. Over-expression of miR-34 family members reduced migration, invasion and cellular proliferation in ovarian cancer cell lines, providing evidence that the loss of miR-34 family members may be involved in the pathobiology of ovarian cancer.

### miRNA-100

miR-100 is a tumor suppressor which has been found to be down-regulated in most of the ovarian cancer cell lines, especially clear cell ovarian carcinoma cell lines and ovarian cancer tissues [83,84]. miR-100 represses mTOR (mammalian target of rapamycin) signaling and increases sensitivity to the cancer drug everolimus (rapamycin analog RAD001) in cell lines derived from clear cell carcinomas. mTOR is a serine/threonine kinase and is a downstream effector of the Akt signaling pathway. mTOR has also been shown to be a possible therapeutic target in both cisplatin-sensitive and cisplatin-resistant clear cell ovarian carcinoma [85]. Low miR-100 expression was associated with shorter overall patient survival and advanced stage ovarian cancer. Moreover, its expression has been shown to be an independent predictor of overall survival in ovarian cancer patients. miR-100 also inhibits the expression of the proto-oncogene PLK1 (Polo-like kinase-1) in ovarian cancer [84].

### miRNA-31

miR-31 is under-expressed in both serous ovarian cancer cell lines and tissues [86]. miRNA-31 inhibits the expression of cell cycle regulators such as E2F2 and STK40, a repressor of p53 mediated transcription, and acts as a tumor suppressor in ovarian cancer. Over expression of miR-31 in ovarian cancer cell lines having non functional p53 pathways lead to decreased proliferation and increased caspase-mediated apoptosis, whereas, over expression of miR-31 had no effect on ovarian cancer cells having wild-type p53 [86]. Thus, miRNA-31 might have therapeutic roles in the case of cancers having p53 mutations.

### miRNA-214/199a*

Up-regulation of miR-214 has been detected in various human malignancies, including pancreatic, prostate, gastric, breast and ovarian cancers as well as malignant melanoma [51,87-90]. miR-214 has extensive roles in chemo-resistance, tumor progression and metastasis [51,87,88,91]. Yang et. al. [51] have shown that miR-214 induces cell survival and cisplatin resistance by targeting PTEN. miRNA microarrays show the aberrant regulation of 36 miRNAs between normal ovarian cells and epithelial ovarian tumors [51]. miR-199a*, miR-214, miR-200a and miR-100 were most highly differentially expressed. miR-199a* and miR-214 were found to be up-regulated in 53 and 56% of the tumor tissues, respectively. miR-214 knockdown was found to abrogate cisplatin resistance in cisplatin-resistant cell line A2780CP, whereas exogenous expression of miR-214 renders cisplatin-sensitive cell line A2780S and OV119 cells resistant to cisplatin induced apoptosis. miR-214 activates the Akt pathway by targeting PTEN, which normally negatively regulates Akt. Constitutive activation of Akt leads to chemo-resistance in different types of tumors including ovarian cancer [92]. Thus, miR-214 possibly plays an important role in cisplatin resistance by targeting the PTEN/Akt pathway. miR-199a and miR-214 have been implicated in the process of differentiation of ovarian cancer stem cells (CSCs) into mature ovarian cancer cells [93]. Twist 1, a transcription factor belonging to basic helix-loop-helix proteins has been shown to regulate the expression of both miR-199a and miR-214 which are part of the human *Dnm3os* gene. Twist 1 is involved in the differentiation of multiple cell lineages, including muscle, cartilage and osteogenic cells [94-97]. Twist 1 levels increase during the differentiation process leading to an increase in miR-199a and miR-214, a decrease in IKKβ expression (target of miR-199a), and a decrease in PTEN expression (target of miR-214). This eventually results in an increase in the pAkt activity leading to the process of differentiation.

### miRNA-376c

miRNA-376c was earlier known as miR-368 and was found to be over expressed in a subset of acute myeloid leukemia [98]. Ye et. al. have shown that miR-376c promotes cell proliferation, survival and spheroid formation in ovarian cancer cells [21]. This is done by suppressing activin receptor-like kinase 7 (ALK7) and its ligand Nodal, which together are able to induce apoptosis in human epithelial ovarian cancer cells. A previous study had demonstrated that the Nodal-ALK7 pathway might be involved in chemosensitivity [99]. miR-376c over expression significantly reduced the effect of cisplatin. Moreover, miR-376c and siRNA inhibitors of Nodal and ALK7 also blocked the effect of carboplatin. Chemosensitive and chemoresistant ovarian tumors showed a differential expression of ALK7 and miR-376c. Immuno-histochemical staining was used to stain tumors with ALK7 and miR-376c was detected using real-time PCR, in patients showing a complete response (CR) and those who had an incomplete response (IR). Patients showing CR showed significant ALK7 staining, whereas the staining intensity was very weak in patients with IR. Additionally, the miR-376c expression level was inversely related to ALK7 in both cases [21].

### miRNA-93

miRNA-93 is part of the miR-106b-25 cluster [100]. It has been shown to promote tumor growth and angiogenesis by targeting integrin-β8 [101]. In ovarian cancer it is up-regulated in cisplatin-resistant ovarian cancer cells [102]. It regulates cisplatin chemosensitivity in cisplatin resistant ovarian cancer cells OVCAR3 and SKOV3 by targeting the phosphatase PTEN. Over-expression of miR-93 in both these cells increased the ratio of phosphorylated Akt/over the total Akt (pAkt/total Akt). Phospho rylated-Akt has been shown to play an important role in multiple drug resistance including cisplatin [92,103].

### miRNA-21

miR-21 is aberrantly expressed and functions as an oncogenic miRNA in many tumors including ovarian cancer [51,104,105]. In ovarian cancer cells it promotes cell proliferation, invasion and migration through targeting PTEN [106]. miR-21 also has a role in resistance to hypoxic conditions which inhibit tumor growth [107]. Protein kinase Akt2 induces miR-21 expression under oxygen deprivation leading to suppression of tumor suppressor proteins PTEN, PDCD4 and Sprouty 1 (targets of miR-21). This results in resistance to hypoxia [107].

### miRNA expression profiles of subtypes of ovarian cancer

In addition to the miRNAs discussed above, a number of studies have profiled the miRNA expression of specific subtypes of ovarian cancer. Nam et. al., used a customized miRNA microarray of 314 human miRNAs to analyze the miRNA expression profiles of serous ovarian cancer tissues as compared to normal ovarian tissues [47]. They found differential expression of 23 miRNAs. miR-21 was most frequently up-regulated and miR-125b was most frequently down- regulated (Table-2). Northern blot analysis confirmed the up-regulation of miR-200c, miR-93 and miR-141 and the down-regulation of let-7b, miR-99a and miR-125b. In a separate study, Li et. al. have shown a negative correlation between miR-22 expression and the metastatic potential in serous ovarian cancer cell lines [108]. Additionally, miR-519a was found to be significantly up-regulated in serous and clear cell carcinomas as compared to the mucinous subtype in tissue samples [109]. Higher expression of miR-519a in late stage serous carcinoma showed positive correlation with poor progression-free survival. miR-153 and miR-485-5p were found to be up-regulated in mucinous ovarian carcinoma. The down-regulation of miR-153 and miR-485-5p showed significant correlation with advanced clinical stage FIGO (International Federation of Gynecology and Obstetrics) grade 3 and miR-519a was found to be high in clinical stages III and IV (advanced clinical stages) as compared to stages I and II (early clinical stages) [109]. As mentioned above miR-100 has been shown to be down-regulated in clear cell ovarian carcinoma cell lines and its over-expression in them inhibited mTOR signaling and enhanced sensitivity to rapamycin analog RAD001 (everolimus) [83] (Table-2). In the same study the authors show the down-regulation of miR-22 and the up-regulation of miR-182 and miR-30a in clear cell ovarian carcinoma cell lines. Over-expression of miR-22 and knockdown of miR-182 could alter the global gene expression pattern of clear cell ovarian cell lines towards a normal state [83].

### miRNAs in ovarian cancer diagnostics / prognostics

Previous studies in ovarian carcinoma have shown that miRNAs can be used in its diagnosis as well as prognosis. Lu et. al. [110] demonstrated that patients having low let-7a-3 methylation had overall worse survival than those with high methylation. The miRNA-200 family plays an important role in ovarian cancer and it has been shown that the miR-200 family cluster, which includes miR-200a, miR-200b and miR-429, can predict poor survival when they are expressed at low levels [111]. Yang et. al. [51] showed that miR-214, miR-199* and miR-200a were associated with high-grade and late stage tumors. In an interesting ovarian cancer study [112] the authors profiled miRNA signatures from tumor-derived exosomes. Levels of 8 miRNAs, which were previously shown to have diagnostic potential (miR-21, 141, 200a, 200c, 200b, 203, 205 and miR-214), were compared in exosomes isolated from serum specimens of women with benign disease and various stages of cancer. The 8 miRNAs had similar expressions between cellular and exosomal miRNAs, with no detection of exosomal miRNAs in control samples. The profile of exosomal miRNAs from ovarian cancer patients was distinctly different from patients with benign disease. HMGA2/let-7 ratio has also been used for prognostic studies [113]. High-mobility group AT-hook 2 (HMGA2), an early embryonic gene is a target of miRNAs let-7a, let-7c and let-7g. Higher HMGA2/let-7 ratio exhibited decreased 5-year progression-free survival (<10%) as compared to a lower ratio (~40%).

### Potential role of miRNAs in ovarian cancer therapeutics

miRNA therapeutics in ovarian cancer can take different forms. Oncogenic miRNAs can be inhibited by using antisense oligonucleotides, antagomirs, sponges or locked nucleic acid (LNA) constructs [114]. Cancer cells have dysregulation in several miRNAs at the same time and targeting a single miRNA is not sufficient for treatment. Multiple-target anti-miRNA antisense oligodeoxyribonucleotide-MTG-AMO (Multiple target-O-modified antagomirs) are used to inhibit multiple miRNAs at the same time [115]. The expression of tumor suppressor miRNAs can be restored by miRNA replacement therapy. Several miRNAs have been used for this purpose. Use of let-7 miRNA mimetics is a potential tool as intra-tumoral delivery of let-7b has been shown to decrease the tumor burden in lung tumors [116,117]. The miR-143-145 cluster has been shown to be frequently deleted in cancer. miR-143 and 145, delivered intravenously to subcutaneous and orthotopic xenografts downregulated the oncogenes RREB1 and KRAS [118]. One of the more beneficial miRNA therapies is miR-34 replacement therapy. P53 protein is known to enhance miR-34 expression and it is mutated in many cancers [119]. The intra-tumoral delivery of miR-34 mimics impaired tumorigenesis on a xenograft model of non-small cell lung cancer, and systemic delivery of miR-34 reduced tumor growth of KrasLSL-G12D^+^ mice [117,120]. Certain small molecule compounds like enoxacin have been shown to restore downregulated miRNAs to a normal miRNA level or expression pattern without affecting normal cells and with no toxicity in *in vivo* models [121,122].

miRNAs can also be used to sensitize tumors to chemotherapy. The efflux of anticancer drugs by ABC transporters is one of the main reasons resistance to chemotherapy drugs develops. miR-9 has been shown to negatively regulate SOX2 which induces the expression of ABC transporters ABCC3 and ABCC6 [123]. Resistance to tamoxifen is restored by the over expression of miR-15 and miR-16. miR-15 and 16 suppress the anti-apoptotic molecule BCL-2 and sensitize the cells to tamoxifen [124]. Similarly, use of antagomirs against miR-21 was found to sensitize cultured cells to the chemotherapeutic agent 5-Fluorouracil (5-FU) [125].

One of the greatest challenges in RNAi therapy continues to be the delivery method of the therapeutic siRNA or miRNA to the target cells. Future focus should be aimed at addressing these issues by engineering an efficient delivery system by use of radiolabeled, tumor specific antibody conjugated nanoformulations to deliver miRNA to the ovarian tumor site. It is very important to formulate an effective delivery method, such as nanotechnology-based delivery approach for microRNA for therapy with the help of strategic image-guided systemic delivery to the tumor. This will be a major contribution in the field of cancer therapeutics and will help overcome challenges in miRNA delivery. The goal of this novel therapeutic approach is to effectively encourage reprogramming of miRNA networks in cancer cells which may lead to a clinically translatable miRNA-based therapy to benefit ovarian cancer patients.

## Conclusions

In spite of all the above advances there is still a long way to go to understand and apply miRNA therapeutics in cancer and ovarian cancer in particular. Identification of unique patterns of deregulated miRNA expression in ovarian cancer provides valuable information that may: serve as molecular biomarkers for tumor diagnosis; identify low and high risk populations of patients, disease prognosis and prevention of cancer, and predict therapeutic responses. One of the areas of improvement in miRNA therapy has been to reduce or eliminate off-target or non-specific effects. This regulatory network is complex because of the fact that a single miRNA can have multiple targets and several miRNAs can have a single target. Therefore, careful designing of therapeutic strategies is needed to overcome these technical issues. Moreover, the identification of new strategies is required to enhance the potency and stability of therapeutic vectors and the specificity of their delivery to tissues. We hope that with increased understanding of the role of miRNAs in cancer development and by designing more efficient miRNA-modulating molecules, miRNA mediated cancer therapy will give a new impetus to the cure for cancer including ovarian cancer.

## Abbreviations

miRNA: MicroRNA; EMT: Epithelial to mesenchymal transition; RISC: RNAinduced silencing complex; UTR: Untranslated region; EOC: Epithelial ovarian cancer/ovarian cancer.

## Competing interests

The authors declare that there are no financial and non financial competing interests.

## Authors’ contributions

MSZ designed and drafted the manuscript; MJ, DMM and SK were involved in the critical revision of the manuscript; SCC gave final approval of the version to be published. All authors read and approved the final manuscript.

## References

[B1] Siegel R Naishadham D Jemal A Cancer statistics, 2012 CA Cancer J Clin 2012 62 1 10 29 10.3322/caac.20138 22237781

[B2] Wright JD Shah M Mathew L Burke WM Culhane J Goldman N Schiff PB Herzog TJ Fertility preservation in young women with epithelial ovarian cancer Cancer 2009 115 18 4118 4126 10.1002/cncr.24461 19670446

[B3] Fung-Kee-Fung M Oliver T Elit L Oza A Hirte HW Bryson P Optimal chemotherapy treatment for women with recurrent ovarian cancer Curr Oncol 2007 14 5 195 208 10.3747/co.2007.148 17938703 PMC2002482

[B4] Takano M Kikuchi Y Yaegashi N Kuzuya K Ueki M Tsuda H Suzuki M Kigawa J Takeuchi S Moriya T Clear cell carcinoma of the ovary: a retrospective multicentre experience of 254 patients with complete surgical staging Br J Cancer 2006 94 10 1369 1374 10.1038/sj.bjc.6603116 16641903 PMC2361284

[B5] du Bois A Luck HJ Meier W Adams HP Mobus V Costa S Bauknecht T Richter B Warm M Schroder W A randomized clinical trial of cisplatin/paclitaxel versus carboplatin/paclitaxel as first-line treatment of ovarian cancer J Natl Cancer Inst 2003 95 17 1320 1329 10.1093/jnci/djg036 12953086

[B6] Shih Ie M Kurman RJ Ovarian tumorigenesis: a proposed model based on morphological and molecular genetic analysis Am J Pathol 2004 164 5 1511 1518 10.1016/S0002-9440(10)63708-X 15111296 PMC1615664

[B7] Morgan RJ Alvarez RD Armstrong DK Burger RA Castells M Chen LM Copeland L Crispens MA Gershenson D Gray H Ovarian Cancer, Version 3.2012 J Natl Compr Canc Netw 2012 10 11 1339 1349 23138163 10.6004/jnccn.2012.0140

[B8] Vergote I du Bois A Amant F Heitz F Leunen K Harter P Neoadjuvant chemotherapy in advanced ovarian cancer: On what do we agree and disagree Gynecol Oncol 2012 Sep 21 (Epub ahead of print). 10.1016/j.ygyno.2012.09.013 23006973

[B9] Gonzalez-Martin A Chiva L Emerging Concerns When Evidence-Based Medicine Is Translated into Real Life: The Case of Neoadjuvant Chemotherapy in Ovarian Cancer Curr Oncol Rep 2012 Oct 9 (epub ahead of print). 10.1007/s11912-012-0278-0 23054938

[B10] Cannistra SA Cancer of the ovary N Engl J Med 2004 351 24 2519 2529 10.1056/NEJMra041842 15590954

[B11] Rabik CA Dolan ME Molecular mechanisms of resistance and toxicity associated with platinating agents Cancer Treat Rev 2007 33 1 9 23 10.1016/j.ctrv.2006.09.006 17084534 PMC1855222

[B12] Fraser M Bai T Tsang BK Akt promotes cisplatin resistance in human ovarian cancer cells through inhibition of p53 phosphorylation and nuclear function Int J Cancer 2008 122 3 534 546 10.1002/ijc.23086 17918180

[B13] Behbakht K Qamar L Aldridge CS Coletta RD Davidson SA Thorburn A Ford HL Six1 overexpression in ovarian carcinoma causes resistance to TRAIL-mediated apoptosis and is associated with poor survival Cancer Res 2007 67 7 3036 3042 10.1158/0008-5472.CAN-06-3755 17409410

[B14] Duan Z Foster R Bell DA Mahoney J Wolak K Vaidya A Hampel C Lee H Seiden MV Signal transducers and activators of transcription 3 pathway activation in drug-resistant ovarian cancer Clin Cancer Res 2006 12 17 5055 5063 10.1158/1078-0432.CCR-06-0861 16951221

[B15] Gan Y Wientjes MG Au JL Expression of basic fibroblast growth factor correlates with resistance to paclitaxel in human patient tumors Pharm Res 2006 23 6 1324 1331 10.1007/s11095-006-0136-6 16741658

[B16] Ferlini C Raspaglio G Cicchillitti L Mozzetti S Prislei S Bartollino S Scambia G Looking at drug resistance mechanisms for microtubule interacting drugs: does TUBB3 work? Curr Cancer Drug Targets 2007 7 8 704 712 10.2174/156800907783220453 18220531

[B17] Bartel DP MicroRNAs: genomics, biogenesis, mechanism, and function Cell 2004 116 2 281 297 10.1016/S0092-8674(04)00045-5 14744438

[B18] Bartels CL Tsongalis GJ MicroRNAs: novel biomarkers for human cancer Clin Chem 2009 55 4 623 631 10.1373/clinchem.2008.112805 19246618

[B19] Sorrentino A Liu CG Addario A Peschle C Scambia G Ferlini C Role of microRNAs in drug-resistant ovarian cancer cells Gynecol Oncol 2008 111 3 478 486 10.1016/j.ygyno.2008.08.017 18823650

[B20] Marchini S Fruscio R Clivio L Beltrame L Porcu L Nerini IF Cavalieri D Chiorino G Cattoretti G Mangioni C Resistance to platinum-based chemotherapy is associated with epithelial to mesenchymal transition in epithelial ovarian cancer Eur J Cancer 2012 Aug 13, (Epub ahead of print). 10.1016/j.ejca.2012.06.026 22897840

[B21] Ye G Fu G Cui S Zhao S Bernaudo S Bai Y Ding Y Zhang Y Yang BB Peng C MicroRNA 376c enhances ovarian cancer cell survival by targeting activin receptor-like kinase 7: implications for chemoresistance J Cell Sci 2011 124 Pt 3 359 368 21224400 10.1242/jcs.072223

[B22] Lee RC Feinbaum RL Ambros V The C. elegans heterochronic gene lin-4 encodes small RNAs with antisense complementarity to lin-14 Cell 1993 75 5 843 854 10.1016/0092-8674(93)90529-Y 8252621

[B23] Wheeler BM Heimberg AM Moy VN Sperling EA Holstein TW Heber S Peterson KJ The deep evolution of metazoan microRNAs Evol Dev 2009 11 1 50 68 10.1111/j.1525-142X.2008.00302.x 19196333

[B24] Ambros V The functions of animal microRNAs Nature 2004 431 7006 350 355 10.1038/nature02871 15372042

[B25] Cai X Hagedorn CH Cullen BR Human microRNAs are processed from capped, polyadenylated transcripts that can also function as mRNAs RNA 2004 10 12 1957 1966 10.1261/rna.7135204 15525708 PMC1370684

[B26] Kim YK Kim VN Processing of intronic microRNAs EMBO J 2007 26 3 775 783 10.1038/sj.emboj.7601512 17255951 PMC1794378

[B27] Pratt AJ MacRae IJ The RNA-induced silencing complex: a versatile gene-silencing machine J Biol Chem 2009 284 27 17897 17901 10.1074/jbc.R900012200 19342379 PMC2709356

[B28] Fabian MR Mathonnet G Sundermeier T Mathys H Zipprich JT Svitkin YV Rivas F Jinek M Wohlschlegel J Doudna JA Mammalian miRNA RISC recruits CAF1 and PABP to affect PABP-dependent deadenylation Mol Cell 2009 35 6 868 880 10.1016/j.molcel.2009.08.004 19716330 PMC2803087

[B29] Grimson A Farh KK Johnston WK Garrett-Engele P Lim LP Bartel DP MicroRNA targeting specificity in mammals: determinants beyond seed pairing Mol Cell 2007 27 1 91 105 10.1016/j.molcel.2007.06.017 17612493 PMC3800283

[B30] Gu S Jin L Zhang F Sarnow P Kay MA Biological basis for restriction of microRNA targets to the 3' untranslated region in mammalian mRNAs Nat Struct Mol Biol 2009 16 2 144 150 10.1038/nsmb.1552 19182800 PMC2713750

[B31] Vasudevan S Tong Y Steitz JA Cell-cycle control of microRNA-mediated translation regulation Cell Cycle 2008 7 11 1545 1549 10.4161/cc.7.11.6018 18469529 PMC2556257

[B32] Lewis BP Shih IH Jones-Rhoades MW Bartel DP Burge CB Prediction of mammalian microRNA targets Cell 2003 115 7 787 798 10.1016/S0092-8674(03)01018-3 14697198

[B33] Flynt AS Lai EC Biological principles of microRNA-mediated regulation: shared themes amid diversity Nat Rev Genet 2008 9 11 831 842 10.1038/nrg2455 18852696 PMC2729318

[B34] Calin GA Dumitru CD Shimizu M Bichi R Zupo S Noch E Aldler H Rattan S Keating M Rai K Frequent deletions and down-regulation of micro- RNA genes miR15 and miR16 at 13q14 in chronic lymphocytic leukemia Proc Natl Acad Sci U S A 2002 99 24 15524 15529 10.1073/pnas.242606799 12434020 PMC137750

[B35] Zhang W Dahlberg JE Tam W MicroRNAs in tumorigenesis: a primer Am J Pathol 2007 171 3 728 738 10.2353/ajpath.2007.070070 17724137 PMC1959494

[B36] Calin GA Ferracin M Cimmino A Di Leva G Shimizu M Wojcik SE Iorio MV Visone R Sever NI Fabbri M A MicroRNA signature associated with prognosis and progression in chronic lymphocytic leukemia N Engl J Med 2005 353 17 1793 1801 10.1056/NEJMoa050995 16251535

[B37] Calin GA Croce CM Chromosomal rearrangements and microRNAs: a new cancer link with clinical implications J Clin Invest 2007 117 8 2059 2066 10.1172/JCI32577 17671640 PMC1934569

[B38] Tagawa H Seto M A microRNA cluster as a target of genomic amplification in malignant lymphoma Leukemia 2005 19 11 2013 2016 10.1038/sj.leu.2403942 16167061

[B39] Calin GA Sevignani C Dumitru CD Hyslop T Noch E Yendamuri S Shimizu M Rattan S Bullrich F Negrini M Human microRNA genes are frequently located at fragile sites and genomic regions involved in cancers Proc Natl Acad Sci U S A 2004 101 9 2999 3004 10.1073/pnas.0307323101 14973191 PMC365734

[B40] Giannakakis A Sandaltzopoulos R Greshock J Liang S Huang J Hasegawa K Li C O'Brien-Jenkins A Katsaros D Weber BL miR-210 links hypoxia with cell cycle regulation and is deleted in human epithelial ovarian cancer Cancer Biol Ther 2008 7 255 264 10.4161/cbt.7.2.5297 18059191 PMC3233968

[B41] Saito Y Liang G Egger G Friedman JM Chuang JC Coetzee GA Jones PA Specific activation of microRNA-127 with downregulation of the proto-oncogene BCL6 by chromatin-modifying drugs in human cancer cells Cancer Cell 2006 9 6 435 443 10.1016/j.ccr.2006.04.020 16766263

[B42] Iorio MV Visone R Di Leva G Donati V Petrocca F Casalini P Taccioli C Volinia S Liu CG Alder H MicroRNA signatures in human ovarian cancer Cancer Res 2007 67 18 8699 8707 10.1158/0008-5472.CAN-07-1936 17875710

[B43] Kumar MS Lu J Mercer KL Golub TR Jacks T Impaired microRNA processing enhances cellular transformation and tumorigenesis Nat Genet 2007 39 5 673 677 10.1038/ng2003 17401365

[B44] He L He X Lim LP de Stanchina E Xuan Z Liang Y Xue W Zender L Magnus J Ridzon D A microRNA component of the p53 tumour suppressor network Nature 2007 447 7148 1130 1134 10.1038/nature05939 17554337 PMC4590999

[B45] Zhang L Volinia S Bonome T Calin GA Greshock J Yang N Liu CG Giannakakis A Alexiou P Hasegawa K Genomic and epigenetic alterations deregulate microRNA expression in human epithelial ovarian cancer Proc Natl Acad Sci U S A 2008 105 19 7004 7009 10.1073/pnas.0801615105 18458333 PMC2383982

[B46] Zhang L Huang J Yang N Greshock J Megraw MS Giannakakis A Liang S Naylor TL Barchetti A Ward MR microRNAs exhibit high frequency genomic alterations in human cancer Proc Natl Acad Sci U S A 2006 103 24 9136 9141 10.1073/pnas.0508889103 16754881 PMC1474008

[B47] Nam EJ Yoon H Kim SW Kim H Kim YT Kim JH Kim JW Kim S MicroRNA expression profiles in serous ovarian carcinoma Clin Cancer Res 2008 14 9 2690 2695 10.1158/1078-0432.CCR-07-1731 18451233

[B48] Dahiya N Sherman-Baust CA Wang TL Davidson B Shih Ie M Zhang Y Becker KG Morin PJ Wood W MicroRNA expression and identification of putative miRNA targets in ovarian cancer PLoS One 2008 3 6 e2436 10.1371/journal.pone.0002436 18560586 PMC2410296

[B49] Wyman SK Parkin RK Mitchell PS Fritz BR O'Briant K Godwin AK Urban N Drescher CW Knudsen BS Tewari M Repertoire of microRNAs in epithelial ovarian cancer as determined by next generation sequencing of small RNA cDNA libraries PLoS One 2009 4 4 e5311 10.1371/journal.pone.0005311 19390579 PMC2668797

[B50] Lee CH Subramanian S Beck AH Espinosa I Senz J Zhu SX Huntsman D van de Rijn M Gilks CB MicroRNA profiling of BRCA1/2 mutation-carrying and non-mutation-carrying high-grade serous carcinomas of ovary PLoS One 2009 4 10 e7314 10.1371/journal.pone.0007314 19798417 PMC2749450

[B51] Yang H Kong W He L Zhao JJ O'Donnell JD Wang J Wenham RM Coppola D Kruk PA Nicosia SV MicroRNA expression profiling in human ovarian cancer: miR-214 induces cell survival and cisplatin resistance by targeting PTEN Cancer Res 2008 68 2 425 433 10.1158/0008-5472.CAN-07-2488 18199536

[B52] Korpal M Lee ES Hu G Kang Y The miR-200 family inhibits epithelial-mesenchymal transition and cancer cell migration by direct targeting of E-cadherin transcriptional repressors ZEB1 and ZEB2 J Biol Chem 2008 283 22 14910 14914 10.1074/jbc.C800074200 18411277 PMC3258899

[B53] Huber MA Kraut N Beug H Molecular requirements for epithelial-mesenchymal transition during tumor progression Curr Opin Cell Biol 2005 17 5 548 558 10.1016/j.ceb.2005.08.001 16098727

[B54] Park SM Gaur AB Lengyel E Peter ME The miR-200 family determines the epithelial phenotype of cancer cells by targeting the E-cadherin repressors ZEB1 and ZEB2 Genes Dev 2008 22 7 894 907 10.1101/gad.1640608 18381893 PMC2279201

[B55] Gregory PA Bert AG Paterson EL Barry SC Tsykin A Farshid G Vadas MA Khew-Goodall Y Goodall GJ The miR-200 family and miR-205 regulate epithelial to mesenchymal transition by targeting ZEB1 and SIP1 Nat Cell Biol 2008 10 5 593 601 10.1038/ncb1722 18376396

[B56] Gregory PA Bracken CP Bert AG Goodall GJ MicroRNAs as regulators of epithelial-mesenchymal transition Cell Cycle 2008 7 20 3112 3118 10.4161/cc.7.20.6851 18927505

[B57] Kong D Li Y Wang Z Banerjee S Ahmad A Kim HR Sarkar FH miR-200 regulates PDGF-D-mediated epithelial-mesenchymal transition, adhesion, and invasion of prostate cancer cells Stem Cells 2009 27 8 1712 1721 10.1002/stem.101 19544444 PMC3400149

[B58] Imai T Horiuchi A Shiozawa T Osada R Kikuchi N Ohira S Oka K Konishi I Elevated expression of E-cadherin and alpha-, beta-, and gamma-catenins in metastatic lesions compared with primary epithelial ovarian carcinomas Hum Pathol 2004 35 12 1469 1476 10.1016/j.humpath.2004.09.014 15619205

[B59] Leskela S Leandro-Garcia LJ Mendiola M Barriuso J Inglada-Perez L Munoz I Martinez-Delgado B Redondo A de Santiago J Robledo M The miR-200 family controls beta-tubulin III expression and is associated with paclitaxel-based treatment response and progression-free survival in ovarian cancer patients Endocr Relat Cancer 2011 18 1 85 95 21051560 10.1677/ERC-10-0148

[B60] Rosell R Scagliotti G Danenberg KD Lord RV Bepler G Novello S Cooc J Crino L Sanchez JJ Taron M Transcripts in pretreatment biopsies from a three-arm randomized trial in metastatic non-small-cell lung cancer Oncogene 2003 22 23 3548 3553 10.1038/sj.onc.1206419 12789263

[B61] Seve P Mackey J Isaac S Tredan O Souquet PJ Perol M Lai R Voloch A Dumontet C Class III beta-tubulin expression in tumor cells predicts response and outcome in patients with non-small cell lung cancer receiving paclitaxel Mol Cancer Ther 2005 4 12 2001 2007 10.1158/1535-7163.MCT-05-0244 16373715

[B62] Seve P Dumontet C Is class III beta-tubulin a predictive factor in patients receiving tubulin-binding agents? Lancet Oncol 2008 9 2 168 175 10.1016/S1470-2045(08)70029-9 18237851

[B63] Koh Y Kim TM Jeon YK Kwon TK Hah JH Lee SH Kim DW Wu HG Rhee CS Sung MW Class III beta-tubulin, but not ERCC1, is a strong predictive and prognostic marker in locally advanced head and neck squamous cell carcinoma Ann Oncol 2009 20 8 1414 1419 10.1093/annonc/mdp002 19468031

[B64] Ferrandina G Zannoni GF Martinelli E Paglia A Gallotta V Mozzetti S Scambia G Ferlini C Class III beta-tubulin overexpression is a marker of poor clinical outcome in advanced ovarian cancer patients Clin Cancer Res 2006 12 9 2774 2779 10.1158/1078-0432.CCR-05-2715 16675570

[B65] Kavallaris M Kuo DY Burkhart CA Regl DL Norris MD Haber M Horwitz SB Taxol-resistant epithelial ovarian tumors are associated with altered expression of specific beta-tubulin isotypes J Clin Invest 1997 100 5 1282 1293 10.1172/JCI119642 9276747 PMC508306

[B66] Mozzetti S Ferlini C Concolino P Filippetti F Raspaglio G Prislei S Gallo D Martinelli E Ranelletti FO Ferrandina G Class III beta-tubulin overexpression is a prominent mechanism of paclitaxel resistance in ovarian cancer patients Clin Cancer Res 2005 11 1 298 305 15671559

[B67] Umezu T Shibata K Kajiyama H Terauchi M Ino K Nawa A Kikkawa F Taxol resistance among the different histological subtypes of ovarian cancer may be associated with the expression of class III beta-tubulin Int J Gynecol Pathol 2008 27 2 207 212 18317222 10.1097/PGP.0b013e318156c838

[B68] Roush S Slack FJ The let-7 family of microRNAs Trends Cell Biol 2008 18 10 505 516 10.1016/j.tcb.2008.07.007 18774294

[B69] Pasquinelli AE Reinhart BJ Slack F Martindale MQ Kuroda MI Maller B Hayward DC Ball EE Degnan B Muller P Conservation of the sequence and temporal expression of let-7 heterochronic regulatory RNA Nature 2000 408 6808 86 89 10.1038/35040556 11081512

[B70] Takamizawa J Konishi H Yanagisawa K Tomida S Osada H Endoh H Harano T Yatabe Y Nagino M Nimura Y Reduced expression of the let-7 microRNAs in human lung cancers in association with shortened postoperative survival Cancer Res 2004 64 11 3753 3756 10.1158/0008-5472.CAN-04-0637 15172979

[B71] Yang N Kaur S Volinia S Greshock J Lassus H Hasegawa K Liang S Leminen A Deng S Smith L MicroRNA microarray identifies Let-7i as a novel biomarker and therapeutic target in human epithelial ovarian cancer Cancer Res 2008 68 24 10307 10314 10.1158/0008-5472.CAN-08-1954 19074899 PMC2762326

[B72] Johnson SM Grosshans H Shingara J Byrom M Jarvis R Cheng A Labourier E Reinert KL Brown D Slack FJ RAS is regulated by the let-7 microRNA family Cell 2005 120 5 635 647 10.1016/j.cell.2005.01.014 15766527

[B73] Bussing I Slack FJ Grosshans H let-7 microRNAs in development, stem cells and cancer Trends Mol Med 2008 14 9 400 409 10.1016/j.molmed.2008.07.001 18674967

[B74] Johnson CD Esquela-Kerscher A Stefani G Byrom M Kelnar K Ovcharenko D Wilson M Wang X Shelton J Shingara J The let-7 microRNA represses cell proliferation pathways in human cells Cancer Res 2007 67 16 7713 7722 10.1158/0008-5472.CAN-07-1083 17699775

[B75] Schultz J Lorenz P Gross G Ibrahim S Kunz M MicroRNA let-7b targets important cell cycle molecules in malignant melanoma cells and interferes with anchorage-independent growth Cell Res 2008 18 5 549 557 10.1038/cr.2008.45 18379589

[B76] Wang Y Hu X Greshock J Shen L Yang X Shao Z Liang S Tanyi JL Sood AK Zhang L Genomic DNA Copy-Number Alterations of the let-7 Family in Human Cancers PLoS One 2012 7 9 e44399 10.1371/journal.pone.0044399 22970210 PMC3435307

[B77] Chen GQ Zhao ZW Zhou HY Liu YJ Yang HJ Systematic analysis of microRNA involved in resistance of the MCF-7 human breast cancer cell to doxorubicin Med Oncol 2010 27 2 406 415 10.1007/s12032-009-9225-9 19412672

[B78] Blower PE Chung JH Verducci JS Lin S Park JK Dai Z Liu CG Schmittgen TD Reinhold WC Croce CM MicroRNAs modulate the chemosensitivity of tumor cells Mol Cancer Ther 2008 7 1 1 9 10.1158/1535-7163.MCT-07-0573 18187804

[B79] Weidhaas JB Babar I Nallur SM Trang P Roush S Boehm M Gillespie E Slack FJ MicroRNAs as potential agents to alter resistance to cytotoxic anticancer therapy Cancer Res 2007 67 23 11111 11116 10.1158/0008-5472.CAN-07-2858 18056433 PMC6070379

[B80] Boyerinas B Park SM Murmann AE Gwin K Montag AG Zillhardt M Hua YJ Lengyel E Peter ME Let-7 modulates acquired resistance of ovarian cancer to Taxanes via IMP-1-mediated stabilization of multidrug resistance 1 Int J Cancer 2012 130 8 1787 1797 10.1002/ijc.26190 21618519 PMC3230767

[B81] Lu L Schwartz P Scarampi L Rutherford T Canuto EM Yu H Katsaros D MicroRNA let-7a: a potential marker for selection of paclitaxel in ovarian cancer management Gynecol Oncol 2011 122 2 366 371 10.1016/j.ygyno.2011.04.033 21571355

[B82] Corney DC Hwang CI Matoso A Vogt M Flesken-Nikitin A Godwin AK Kamat AA Sood AK Ellenson LH Hermeking H Frequent downregulation of miR-34 family in human ovarian cancers Clin Cancer Res 2010 16 4 1119 1128 10.1158/1078-0432.CCR-09-2642 20145172 PMC2822884

[B83] Nagaraja AK Creighton CJ Yu Z Zhu H Gunaratne PH Reid JG Olokpa E Itamochi H Ueno NT Hawkins SM A link between mir-100 and FRAP1/mTOR in clear cell ovarian cancer Mol Endocrinol 2010 24 2 447 463 10.1210/me.2009-0295 20081105 PMC2817607

[B84] Peng DX Luo M Qiu LW He YL Wang XF Prognostic implications of microRNA-100 and its functional roles in human epithelial ovarian cancer Oncol Rep 2012 27 4 1238 1244 22246341 10.3892/or.2012.1625 PMC3583406

[B85] Mabuchi S Kawase C Altomare DA Morishige K Sawada K Hayashi M Tsujimoto M Yamoto M Klein-Szanto AJ Schilder RJ mTOR is a promising therapeutic target both in cisplatin-sensitive and cisplatin-resistant clear cell carcinoma of the ovary Clin Cancer Res 2009 15 17 5404 5413 10.1158/1078-0432.CCR-09-0365 19690197 PMC2743856

[B86] Creighton CJ Fountain MD Yu Z Nagaraja AK Zhu H Khan M Olokpa E Zariff A Gunaratne PH Matzuk MM Molecular profiling uncovers a p53-associated role for microRNA-31 in inhibiting the proliferation of serous ovarian carcinomas and other cancers Cancer Res 2010 70 5 1906 1915 10.1158/0008-5472.CAN-09-3875 20179198 PMC2831102

[B87] Ueda T Volinia S Okumura H Shimizu M Taccioli C Rossi S Alder H Liu CG Oue N Yasui W Relation between microRNA expression and progression and prognosis of gastric cancer: a microRNA expression analysis Lancet Oncol 2010 11 2 136 146 10.1016/S1470-2045(09)70343-2 20022810 PMC4299826

[B88] Penna E Orso F Cimino D Tenaglia E Lembo A Quaglino E Poliseno L Haimovic A Osella-Abate S De Pitta C microRNA-214 contributes to melanoma tumour progression through suppression of TFAP2C EMBO J 2011 30 10 1990 2007 10.1038/emboj.2011.102 21468029 PMC3098476

[B89] Volinia S Calin GA Liu CG Ambs S Cimmino A Petrocca F Visone R Iorio M Roldo C Ferracin M A microRNA expression signature of human solid tumors defines cancer gene targets Proc Natl Acad Sci U S A 2006 103 7 2257 2261 10.1073/pnas.0510565103 16461460 PMC1413718

[B90] Blenkiron C Goldstein LD Thorne NP Spiteri I Chin SF Dunning MJ Barbosa-Morais NL Teschendorff AE Green AR Ellis IO MicroRNA expression profiling of human breast cancer identifies new markers of tumor subtype Genome Biol 2007 8 10 R214 10.1186/gb-2007-8-10-r214 17922911 PMC2246288

[B91] Qiang R Wang F Shi LY Liu M Chen S Wan HY Li YX Li X Gao SY Sun BC Plexin-B1 is a target of miR-214 in cervical cancer and promotes the growth and invasion of HeLa cells Int J Biochem Cell Biol 2011 43 4 632 641 10.1016/j.biocel.2011.01.002 21216304

[B92] Testa JR Bellacosa A AKT plays a central role in tumorigenesis Proc Natl Acad Sci U S A 2001 98 20 10983 10985 10.1073/pnas.211430998 11572954 PMC58668

[B93] Yin G Chen R Alvero AB Fu HH Holmberg J Glackin C Rutherford T Mor G TWISTing stemness, inflammation and proliferation of epithelial ovarian cancer cells through MIR199A2/214 Oncogene 2010 29 24 3545 3553 10.1038/onc.2010.111 20400975 PMC2889129

[B94] Lee MS Lowe GN Strong DD Wergedal JE Glackin CA TWIST, a basic helix-loop-helix transcription factor, can regulate the human osteogenic lineage J Cell Biochem 1999 75 4 566 577 10.1002/(SICI)1097-4644(19991215)75:4<566::AID-JCB3>3.0.CO;2-0 10572240

[B95] Lee MS Lowe G Flanagan S Kuchler K Glackin CA Human Dermo-1 has attributes similar to twist in early bone development Bone 2000 27 5 591 602 10.1016/S8756-3282(00)00380-X 11062344

[B96] Bialek P Kern B Yang X Schrock M Sosic D Hong N Wu H Yu K Ornitz DM Olson EN A twist code determines the onset of osteoblast differentiation Dev Cell 2004 6 3 423 435 10.1016/S1534-5807(04)00058-9 15030764

[B97] Ota MS Loebel DA O'Rourke MP Wong N Tsoi B Tam PP Twist is required for patterning the cranial nerves and maintaining the viability of mesodermal cells Dev Dyn 2004 230 2 216 228 10.1002/dvdy.20047 15162501

[B98] Li Z Lu J Sun M Mi S Zhang H Luo RT Chen P Wang Y Yan M Qian Z Distinct microRNA expression profiles in acute myeloid leukemia with common translocations Proc Natl Acad Sci U S A 2008 105 40 15535 15540 10.1073/pnas.0808266105 18832181 PMC2563085

[B99] Xu G Zhong Y Munir S Yang BB Tsang BK Peng C Nodal induces apoptosis and inhibits proliferation in human epithelial ovarian cancer cells via activin receptor-like kinase 7 J Clin Endocrinol Metab 2004 89 11 5523 5534 10.1210/jc.2004-0893 15531507

[B100] Li Y Tan W Neo TW Aung MO Wasser S Lim SG Tan TM Role of the miR-106b-25 microRNA cluster in hepatocellular carcinoma Cancer Sci 2009 100 7 1234 1242 10.1111/j.1349-7006.2009.01164.x 19486339

[B101] Fang L Deng Z Shatseva T Yang J Peng C Du WW Yee AJ Ang LC He C Shan SW MicroRNA miR-93 promotes tumor growth and angiogenesis by targeting integrin-beta8 Oncogene 2011 30 7 806 821 10.1038/onc.2010.465 20956944

[B102] Fu X Tian J Zhang L Chen Y Hao Q Involvement of microRNA-93, a new regulator of PTEN/Akt signaling pathway, in regulation of chemotherapeutic drug cisplatin chemosensitivity in ovarian cancer cells FEBS Lett 2012 586 9 1279 1286 10.1016/j.febslet.2012.03.006 22465665

[B103] Altomare DA Wang HQ Skele KL De Rienzo A Klein-Szanto AJ Godwin AK Testa JR AKT and mTOR phosphorylation is frequently detected in ovarian cancer and can be targeted to disrupt ovarian tumor cell growth Oncogene 2004 23 34 5853 5857 10.1038/sj.onc.1207721 15208673

[B104] Si ML Zhu S Wu H Lu Z Wu F Mo YY miR-21-mediated tumor growth Oncogene 2007 26 19 2799 2803 10.1038/sj.onc.1210083 17072344

[B105] Slaby O Svoboda M Fabian P Smerdova T Knoflickova D Bednarikova M Nenutil R Vyzula R Altered expression of miR-21, miR-31, miR-143 and miR-145 is related to clinicopathologic features of colorectal cancer Oncology 2007 72 5–6 397 402 18196926 10.1159/000113489

[B106] Lou Y Yang X Wang F Cui Z Huang Y MicroRNA-21 promotes the cell proliferation, invasion and migration abilities in ovarian epithelial carcinomas through inhibiting the expression of PTEN protein Int J Mol Med 2010 26 6 819 827 21042775 10.3892/ijmm_00000530

[B107] Polytarchou C Iliopoulos D Hatziapostolou M Kottakis F Maroulakou I Struhl K Tsichlis PN Akt2 regulates all Akt isoforms and promotes resistance to hypoxia through induction of miR-21 upon oxygen deprivation Cancer Res 2011 71 13 4720 4731 10.1158/0008-5472.CAN-11-0365 21555366 PMC3129420

[B108] Li J Liang S Yu H Zhang J Ma D Lu X An inhibitory effect of miR-22 on cell migration and invasion in ovarian cancer Gynecol Oncol 2010 119 3 543 548 10.1016/j.ygyno.2010.08.034 20869762

[B109] Kim TH Kim YK Kwon Y Heo JH Kang H Kim G An HJ Deregulation of miR-519a, 153, and 485-5p and its clinicopathological relevance in ovarian epithelial tumours Histopathology 2010 57 5 734 743 10.1111/j.1365-2559.2010.03686.x 21083603

[B110] Lu L Katsaros D de la Longrais IA Sochirca O Yu H Hypermethylation of let-7a-3 in epithelial ovarian cancer is associated with low insulin-like growth factor-II expression and favorable prognosis Cancer Res 2007 67 21 10117 10122 10.1158/0008-5472.CAN-07-2544 17974952

[B111] Hu X Macdonald DM Huettner PC Feng Z El Naqa IM Schwarz JK Mutch DG Grigsby PW Powell SN Wang X A miR-200 microRNA cluster as prognostic marker in advanced ovarian cancer Gynecol Oncol 2009 114 3 457 464 10.1016/j.ygyno.2009.05.022 19501389

[B112] Taylor DD Gercel-Taylor C MicroRNA signatures of tumor-derived exosomes as diagnostic biomarkers of ovarian cancer Gynecol Oncol 2008 110 1 13 21 10.1016/j.ygyno.2008.04.033 18589210

[B113] Shell S Park SM Radjabi AR Schickel R Kistner EO Jewell DA Feig C Lengyel E Peter ME Let-7 expression defines two differentiation stages of cancer Proc Natl Acad Sci U S A 2007 104 27 11400 11405 10.1073/pnas.0704372104 17600087 PMC2040910

[B114] Garzon R Marcucci G Croce CM Targeting microRNAs in cancer: rationale, strategies and challenges Nat Rev Drug Discov 2010 9 10 775 789 10.1038/nrd3179 20885409 PMC3904431

[B115] Lu Y Xiao J Lin H Bai Y Luo X Wang Z Yang B A single anti-microRNA antisense oligodeoxyribonucleotide (AMO) targeting multiple microRNAs offers an improved approach for microRNA interference Nucleic Acids Res 2009 37 3 e24 10.1093/nar/gkn1053 19136465 PMC2647303

[B116] Trang P Medina PP Wiggins JF Ruffino L Kelnar K Omotola M Homer R Brown D Bader AG Weidhaas JB Regression of murine lung tumors by the let-7 microRNA Oncogene 2010 29 11 1580 1587 10.1038/onc.2009.445 19966857 PMC2841713

[B117] Trang P Wiggins JF Daige CL Cho C Omotola M Brown D Weidhaas JB Bader AG Slack FJ Systemic delivery of tumor suppressor microRNA mimics using a neutral lipid emulsion inhibits lung tumors in mice Mol Ther 2011 19 6 1116 1122 10.1038/mt.2011.48 21427705 PMC3129804

[B118] Pramanik D Campbell NR Karikari C Chivukula R Kent OA Mendell JT Maitra A Restitution of tumor suppressor microRNAs using a systemic nanovector inhibits pancreatic cancer growth in mice Mol Cancer Ther 2011 10 8 1470 1480 10.1158/1535-7163.MCT-11-0152 21622730 PMC3154495

[B119] Suzuki HI Yamagata K Sugimoto K Iwamoto T Kato S Miyazono K Modulation of microRNA processing by p53 Nature 2009 460 7254 529 533 10.1038/nature08199 19626115

[B120] Liu C Kelnar K Liu B Chen X Calhoun-Davis T Li H Patrawala L Yan H Jeter C Honorio S The microRNA miR-34a inhibits prostate cancer stem cells and metastasis by directly repressing CD44 Nat Med 2011 17 2 211 215 10.1038/nm.2284 21240262 PMC3076220

[B121] Melo S Villanueva A Moutinho C Davalos V Spizzo R Ivan C Rossi S Setien F Casanovas O Simo-Riudalbas L Small molecule enoxacin is a cancer-specific growth inhibitor that acts by enhancing TAR RNA-binding protein 2-mediated microRNA processing Proc Natl Acad Sci U S A 2011 108 11 4394 4399 10.1073/pnas.1014720108 21368194 PMC3060242

[B122] Shan G Li Y Zhang J Li W Szulwach KE Duan R Faghihi MA Khalil AM Lu L Paroo Z A small molecule enhances RNA interference and promotes microRNA processing Nat Biotechnol 2008 26 8 933 940 10.1038/nbt.1481 18641635 PMC2831467

[B123] Jeon HM Sohn YW Oh SY Kim SH Beck S Kim S Kim H ID4 imparts chemoresistance and cancer stemness to glioma cells by derepressing miR-9*-mediated suppression of SOX2 Cancer Res 2011 71 9 3410 3421 10.1158/0008-5472.CAN-10-3340 21531766

[B124] Cittelly DM Das PM Salvo VA Fonseca JP Burow ME Jones FE Oncogenic HER2{Delta}16 suppresses miR-15a/16 and deregulates BCL-2 to promote endocrine resistance of breast tumors Carcinogenesis 2010 31 12 2049 2057 10.1093/carcin/bgq192 20876285 PMC2994280

[B125] Hwang JH Voortman J Giovannetti E Steinberg SM Leon LG Kim YT Funel N Park JK Kim MA Kang GH Identification of microRNA-21 as a biomarker for chemoresistance and clinical outcome following adjuvant therapy in resectable pancreatic cancer PLoS One 2010 5 5 e10630 10.1371/journal.pone.0010630 20498843 PMC2871055

